# Dynamic covariation between gene expression and proteome characteristics

**DOI:** 10.1186/1471-2105-6-215

**Published:** 2005-08-30

**Authors:** Mansour Taghavi Azar Sharabiani, Markku Siermala, Tommi O Lehtinen, Mauno Vihinen

**Affiliations:** 1 Institute of Medical Technology, FI-33014 University of Tampere, Finland; 2 Research Unit, Tampere University Hospital, FI-33520 Tampere, Finland

## Abstract

**Background:**

Cells react to changing intra- and extracellular signals by dynamically modulating complex biochemical networks. Cellular responses to extracellular signals lead to changes in gene and protein expression. Since the majority of genes encode proteins, we investigated possible correlations between protein parameters and gene expression patterns to identify proteome-wide characteristics indicative of trends common to expressed proteins.

**Results:**

Numerous bioinformatics methods were used to filter and merge information regarding gene and protein annotations. A new statistical time point-oriented analysis was developed for the study of dynamic correlations in large time series data. The method was applied to investigate microarray datasets for different cell types, organisms and processes, including human B and T cell stimulation, *Drosophila melanogaster *life span, and *Saccharomyces cerevisiae *cell cycle.

**Conclusion:**

We show that the properties of proteins synthesized correlate dynamically with the gene expression profile, indicating that not only is the actual identity and function of expressed proteins important for cellular responses but that several physicochemical and other protein properties correlate with gene expression as well. Gene expression correlates strongly with amino acid composition, composition- and sequence-derived variables, functional, structural, localization and gene ontology parameters. Thus, our results suggest that a dynamic relationship exists between proteome properties and gene expression in many biological systems, and therefore this relationship is fundamental to understanding cellular mechanisms in health and disease.

## Background

Cells react to changing intra- and extracellular signals by dynamically modulating complex biochemical networks, and cellular responses to extracellular signals lead to changes in gene and protein expression. These processes can be monitored using genomics and proteomics methods. A number of supervised and unsupervised clustering techniques are routinely applied to classify and group genes based on their expression profiles [1]. While these approaches are sufficient for a general grouping of genes, they do not explain why various genes are coexpressed or whether different regulatory mechanisms are involved. Some studies have focused on the properties of coexpressed genes, such as chromosomal location [2-4], regulatory regions and promoters [5,6]. Correlations have also been observed for some of the properties of encoded proteins, such as function and classification of expressed proteins, including those annotated in MIPS [7,8], gene ontologies [9-11], and structural classes [7]. Furthermore, proteins encoded by coexpressed genes are more likely to interact than proteins in general [12,13].

Since the majority of genes encode proteins, we investigated possible correlations between protein-related properties and gene expression patterns to identify proteome-wide features indicative of trends common to expressed proteins. For example, because the cytoplasm, nucleus and extracellular space have different physicochemical properties, such as pH, ionic composition, and protein concentration, the properties of the proteins that are targeted to different cellular compartments are also different. Because there is variation in the specific proteins that comprise the various proteomes, it is intriguing to hypothesize that cellular signaling leads to significant changes in the protein properties of cells. This idea is supported by studies of the relationship between the overall properties of proteins and their amino acid composition, which has been correlated with protein surface properties [14], subcellular localization [15-17], protein structural class [18], and thermal stability [19].

## Results and discussion

A number of microarray datasets for several different cell types and organisms were analyzed to study possible transcriptome-proteome correlations. Expression studies have revealed certain correlations between genome-related features and coexpressed genes, including co-localization [2-4] and the conservation of 5' regions containing regulatory sequences [5,6]. Cells respond to changes in intra- or extracellular environment by altering gene expression to produce proteins that are appropriate for the response. Here we applied the Spearman linear correlation to monitor covariations between a number of proteome parameters and gene expression levels along a time series. We observed very significant and dynamic correlations in all the datasets we investigated. We investigated several high-quality datasets for different cell types, treatments, and organisms, including human T cell stimulation [20] and B cell stimulation datasets [8,21], yeast cell cycle data [22], and *Drosophila melanogaster *life cycle data [23].

T cell receptor (TCR) activation on the surface of T cells is essential for mounting an adaptive immune response against viruses and microbes. The TCR is a multiprotein complex that activates a large number of signaling pathways [24]. Both the CD3 subunit and a co-receptor such as CD28 must be engaged for optimal activation [25]. Microarray analysis at the transcriptome level has revealed changes in the expression of a large number of TCR-related genes [20]. We have now developed a robust method to identify significant correlations between gene expression levels and 114 protein properties over six time points following TCR activation. We found that amino acid composition and several other protein properties covary with gene expression. These results indicate, for the first time, that gene expression profiles and the properties of the encoded proteins have an integral and dynamic relationship and that the protein constituents and overall properties of the proteome are tightly linked and regulated.

### Time point-oriented analysis

**Figure 1:**
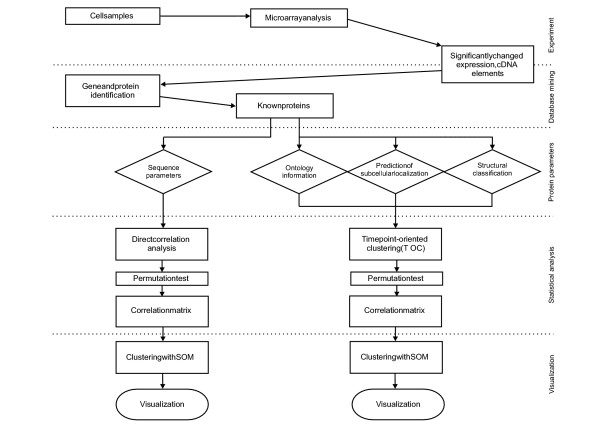
**Schematic overview of the analysis**. Schema for data mining and statistical analysis. Correlation analysis at each time point was carried out between calculated protein parameters and gene expression levels. In the time point-oriented clustering (TOC) at each time point, correlations were analyzed between the medians of expression levels of the clusters and the medians of the proportions of variables for corresponding proteins.

To study transcriptome-proteome correlations, we introduced time point-oriented analysis. Protein sequence-derived parameters were correlated to gene expression separately at each time point. Time point-oriented clustering (TOC), in which the expression of genes at each time point is clustered separately, was used for other features. Fig. 1 presents the general overview of the strategy for the statistical analysis. For sequence-based parameters, direct correlation with gene expression levels was calculated. For other protein features, genes were organized into clusters separately in each time point with TOC after which correlation of the expression and protein parameters was calculated. Following TOC, the Spearman correlation was used to test linear correlations between the median of gene expression level and the protein variables in the corresponding clusters. Due to the nature and scarceness of functional and structural data, it was necessary to cluster the data prior to conducting the correlation test. Moreover, we were able to compare the correlation coefficients by unifying the sample sizes using a fixed number of clusters (20) for each data type. The resulting correlation matrix was clustered to group the data based on the protein characteristics, and then the results were visualized. See Methods for a detailed description.

### Correlation between transcriptome and proteomes

**Figure 2:**
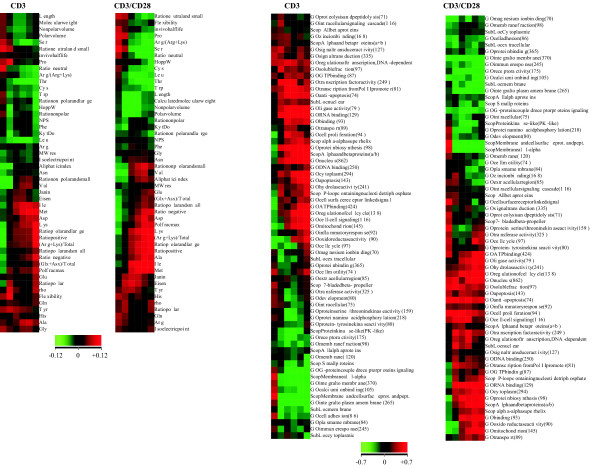
**Correlation analysis of the dataset for stimulated T cells**. Visualization of Spearman's linear correlations between gene expression and protein properties in T cells stimulated with CD3 (left) or costimulated with CD3/CD28 (right). The columns represent time points 1 to 6 corresponding to 1, 2, 6, 12, 24 and 48 hr after stimulation. The 114 variables (rows) consist of 20 amino acid proportions described by three-letter abbreviations, 29 sequence- or amino acid composition-derived parameters (panels to the left), 50 gene ontologies (marked by GO at the beginning), four predicted subcellular localizations (specified by SubLoc), and 11 structural parameters from SCOP (panels to the right). Correlation coefficients are color-coded. Red indicates a positive correlation; i.e., increased production of proteins, which is either attributed to a property (within the categories of GO, SCOP, or SubLoc) or to higher proportions of a property (including amino acids and physicochemical parameters), leading to relative enrichment of that property. Green indicates a negative correlation; i.e., reduced production of proteins, which is either attributed to the property or to higher proportions of the property, leading to relative depletion of that property. The magnitude of the correlation coefficients is represented by color intensity, as indicated at the bottom. The range is from -0.12 to 0.12 for sequence-derived parameters and -0.7 to 0.7 for categorical data used in the TOC approach. The numbers in brackets indicate the genes for which gene ontologies, subcellular localization or structural classes were identified. The correlation coefficients are clustered using the SOM method so as to group features manifesting similar patterns.

**Figure 3:**
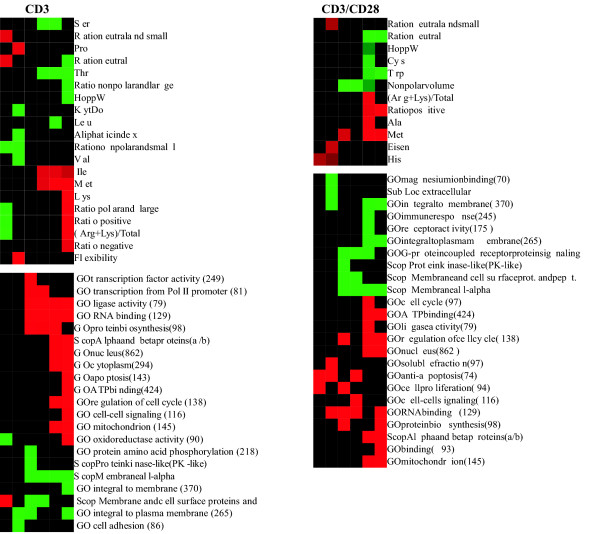
**The most statistically significant observations for CD3-stimulated (left) and CD3/CD28-costimulated (right) datasets**. Only those features with p ≤ 0.05 for both the correlation coefficient and permutation test are shown. Features are organized by SOM clustering. Sequence-derived parameters are on top, with the others below. Color-coding is as in Fig. 2. The numbers in brackets indicate the genes for which gene ontologies, subcellular localization or structural classes were identified. Amino acids composition parameters were calculated for all the proteins. The actual number of proteins used in any individual analysis varied due to different amounts of missing data in different experiments and at different time points.

The 114 variables at six time points in two data sets (CD3 and CD3/CD28 costimulation) yielded 1,368 correlation tests. The huge amount of information was organized according to time points and variables, clustered using the Self Organizing Map (SOM) method and visualized as in Figs. 2 and 3. A total of 177 results (13% of the tests) were statistically significant (with a correlation coefficient either ≥ 0.12 or ≤ (-0.12) for sequence-derived parameters, or ≥ 0.7 or ≤ (-0.7) with TOC). The p value for the correlation study and for the permutation test had to be ≤ 0.05. When considering the large numbers of genes in the datasets, the correlation coefficient value of 0.12 is notable especially since the results were statistically significant based on the p values of both the correlation coefficients and permutation tests of 1,000 rounds. The results indicate clear trends and correlations at subsequent time points as well. It is unlikely that the properties of all the proteins would correlate with gene expression; still the increased/decreased production of certain kinds of proteins is very clear and statistically significant.

**Figure 4:**
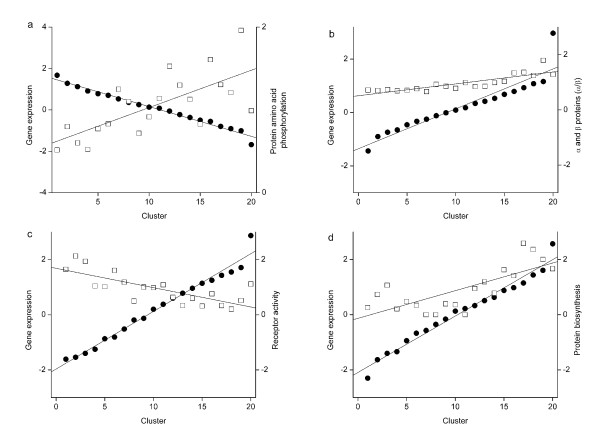
**Examples of correlations between gene expression and proteome parameters**. Comparison of the medians of gene expression (●) and medians of the observed/expected ratio of proteins associated with functional and structural variables (□). **a**, Protein amino acid phosphorylation, time point 3 in the CD3 dataset; **b**, SCOP classification of α/β proteins, time point 6 in the CD3 dataset; **c**, Gene ontology for receptor activity, time point 5 in the CD3/CD28-costimulated dataset; and **d**, Gene ontology for protein biosynthesis, time point 3 in the CD3/CD28-costimulated dataset. The graphs indicate both positive and negative correlations. The clusters are arranged so that the medians of gene expression along the time points are in descending order.

Of particular interest are the characteristics that indicated trends in more than one data set or at more than one time point. Of the 75 and 102 significant results in the CD3 stimulation and CD3/CD28 costimulation experiments, respectively, 44 (25%) were common to both (Fig. 3). Not a single parameter showed opposite correlation between the two experiments, indicating that CD28 activation mainly strengthens the signaling downstream of CD3 stimulation. Very early and late time points after CD3 or costimulation generally yielded stronger correlations. Some examples of these correlations are shown in Fig. 4.

Subcellular localization data were obtained from three individual sources: gene ontology [26], SCOP [27], and prediction. The consistency of the results from the three independent sources reflects the overall high quality of the analysis (Figs. 2 and 3). Membrane proteins showed a negative correlation; i.e., they are relatively underrepresented according to each of the three sources, although the extent of significance varies.

Amino acid frequencies for about half of the residues correlated with the level of gene expression. Lysine dominated the (R+K)/total parameter, correlating positively in both the CD3 and CD3/CD28 costimulation datasets. The aliphatic index, which measures the proportion of aliphatic residues (A, I, L, and V), correlated negatively in the CD3 dataset. The individual aliphatic residues, however, generally gave positive correlations, with the exception of L and V. The frequency of S and T correlated negatively with gene expression in both datasets. The data also suggest that aromatic residues do not generally covary with gene expression. The ratio of polar and large, negative, positive, as well as nonpolar and small residues correlated positively in both datasets. The effect was stronger in the costimulation dataset. Nonpolar residues correlated negatively. Overall hydropathy parameters correlated negatively with expression levels, although this type of correlation varied across time points, due most likely to differences in the parameters contributing to the hydropathy scales. The metabolic cost of amino acid production has been previously evaluated; e.g., for *Escherichia coli *[28]. However, the number of high-energy phosphate bonds required did not correlate with the results shown in Fig. 3. The most costly residues, W, F and Y, were not underrepresented, and the least costly amino acids, G and A (except for A at two time points in CD3/CD28 data), were not enriched.

Of particular interest are the characteristics that yielded negative correlations at early time points and positive correlations at late time points, and vice versa. The trend is from a negative towards a positive correlation for the ratio of positive amino acids and (R+K)/total parameters in the CD3 dataset, and D in the costimulation dataset. Serine showed the opposite trend in the CD3 dataset. The proportion of neutral residues and oxidoreductase activity correlated negatively at early time points, but only in the CD3 dataset. The ratio of nonpolar and small amino acids correlated negatively at early time points in the CD3 dataset and positively only at late time points in the costimulation dataset. These results clearly indicate a strong correlation between gene expression and the amino acid composition of protein products. In particular, polar and charged residues correlate strongly, and there is a biased occurrence of hydrophobic and aliphatic residues. Interestingly, leucine and isoleucine, which are hydrophobic structural isomers, had opposite correlations.

Protein molecular size and weight are weak indicators, because only MWRES (molecular weight per residue) correlated significantly in the costimulation dataset. Of the volume-related parameters, nonpolar and polar volume correlated negatively, and Polfrac max correlated positively in costimulated cells. Protein flexibility correlated positively in the CD3 dataset at one time point. The most flexible residues, G and A, did not correlate with the transcriptome (except for A at two time points).

SCOP [27] contains information about classification of protein structures at four levels. In addition to membrane and cell surface proteins and peptides, intracellular proteins correlated positively with gene expression in the CD3/CD28 costimulation dataset.

**Figure 5:**
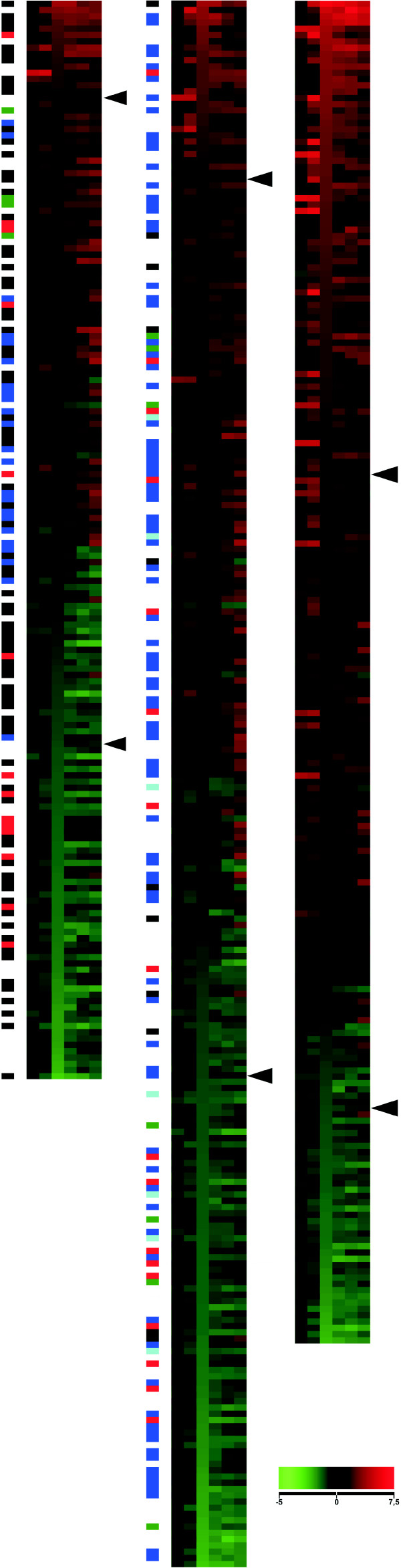
**Gene expression in enriched Gene Ontology classes in the T cell dataset**. The left array shows the gene ontology group receptor activity (4872), the middle array shows protein amino acid phosphorylation (6468), and the right array shows transcription factor activity (3700). Genes and proteins were annotated extensively. Signaling activities in the first two ontology classes are indicated by color coding of the block to the left of the expression patterns: protein tyrosine kinase (PTK, red), protein serine/threonine kinase (PSK, blue), dual-specificity kinase (DSK, cyan), receptor (R, black), and protein phosphatase (green). Genes are sorted based on their expression at time point 3. The box "◀" indicates the boundaries for significantly overexpressed, unchanged, and underexpressed genes at time point 3. Only 9% of receptor activity genes are significantly overexpressed, whereas 31% are significantly suppressed. All PSKs are located in the middle (i.e., changes in their gene expression at time point 3 are insignificant). Hypergeometric analysis revealed significant overrepresentation (p < 0.0008) of PSKs as well as significant underrepresentation of PTKs and Rs in this group. PTKs are overrepresented among the underexpressed genes (p < 0.005). When the genes are grouped according to the significance of their expression at time point 6, very significant over- and underrepresentation of PSKs is apparent among the groups of over- and underexpressed genes, respectively. A similar hypergeometric analysis for protein amino acid phosphorylation indicates consistently significant PSK underrepresentation and PTK overrepresentation among underexpressed genes at time point 3 and relative PSK enrichment and R depletion at time point 6. Significant depletion of membrane proteins and those integral to the plasma membrane appears at late time points (Figs. 2 and 3).

Fifty ontology classes had a large number of entries, 26 of which correlated significantly with expression (Fig. 3). Many of the ontologies were related either to signal transduction or subcellular localization (9 and 11 ontology groups, respectively), with 23 significant findings altogether. Surprisingly, ontologies for immunological or inflammatory responses were not significantly enriched. In fact, the ontology for immune response correlated negatively at time point 5 in the coexpression dataset. Fig. 5 shows the gene expression patterns for some of the largest ontologies.

Intracellular proteins correlated positively towards the end of the time series in both datasets. Membrane proteins in general and those integral to the plasma membrane were significantly underrepresented at several time points. Based on the prediction data, extracellular proteins correlated negatively with gene expression in the CD3/CD28 dataset.

Our results are somewhat related to previous analyses of the yeast proteomes [7,29], data from which indicate that the abundance of some amino acids and certain overall structural and functional properties correlate with protein abundance. However, no statistically significant differences were observed when studying time points in the diauxic shift data [7]. Some of the yeast results [7,29] do not agree with the T cell data (e.g., the enrichment of amino acids). Lymphocytes function as individual cells, and in this respect are similar to unicellular organisms like yeast; however, lymphocyte function and development are critically linked to the presence and activities of other cells. Of the observations made in yeast, only the enrichment of lysine agrees with our results. Some of the differences are undoubtedly due to the different organisms used in these studies, whereas others may have arisen from differences in experimental goals and methods.

The previous yeast study [29] was aimed at calculating the abundance of protein products; however, we did not attempt such calculations due to the lack of experimental data as well as the problems inherent in correlating protein abundance with that of mRNAs [30,31].

### T cell signaling

TCR stimulation activates several signaling pathways [24,32,33], and the detailed annotations of the genes having prominent changes in expression that were generated by our analyses facilitated the identification of these processes and pathways. Receptor activity had a significant negative correlation at time point 5 in the costimulation dataset. There were only a few activated receptors, including IgG receptor, IL-7 receptor, some forms of TNF receptor and G protein-coupled receptors (GPCRs) (Fig. 5). Another significantly affected ontology group was protein amino acid phosphorylation (time point 3 in the CD3 experiment), which was the most characteristic property of the numerous signaling-related observations. There were few changes in signaling molecules in the first hour after stimulation. Several known components of the TCR signaling pathway were overexpressed, including those of the MAP kinase, Ca^2+^-related signaling, and NF-κB pathways. Subsequently, transcription factors became activated, and a significant positive correlation was observed at time point 3 in CD3-stimulated cells. The enriched transcription factors included early growth response factors 1, 2 and 4, NFAT, NF-κB, Jun, Fos, B cell translocation gene, and interferon regulatory factor. Thus, all the major transcription factor components active in TCR signaling were present (i.e., Fos and Jun forming AP-1, NFAT, and NF-κB). In agreement with these results, transcription factors expressed in lymphocytes have highly regulated expression patterns [21].

We analyzed a number of additional datasets to address whether the associations seen in the T cell data analysis represent sporadic observations that are cell type-specific. Dynamic covariation occurred between gene expression profiles and a number of proteome characteristics describing amino acid proportions and physicochemical properties in all the investigated cell types and organisms, suggesting that our T cell data is representative of cells in general.

### Human B cell data analysis

The B cell dataset [8,21] represents genes involved in maturation in anti-immunoglobulin M-stimulated Ramos cells. The development of adaptive immunity and responses to foreign molecules and organisms is based on the highly regulated production of hundreds of proteins. B cell maturation is a multi-step process that requires the ordered expression of a large number of genes. B cell differentiation is activated by non-covalent cross-linking of the B cell receptor (BCR), initiating cellular signaling cascades that ultimately activate nuclear transcription factors. Then, transcriptional activation represses or activates gene expression leading to B cell proliferation, upregulation of surface activation markers, and increased antibody synthesis.

**Figure 6:**
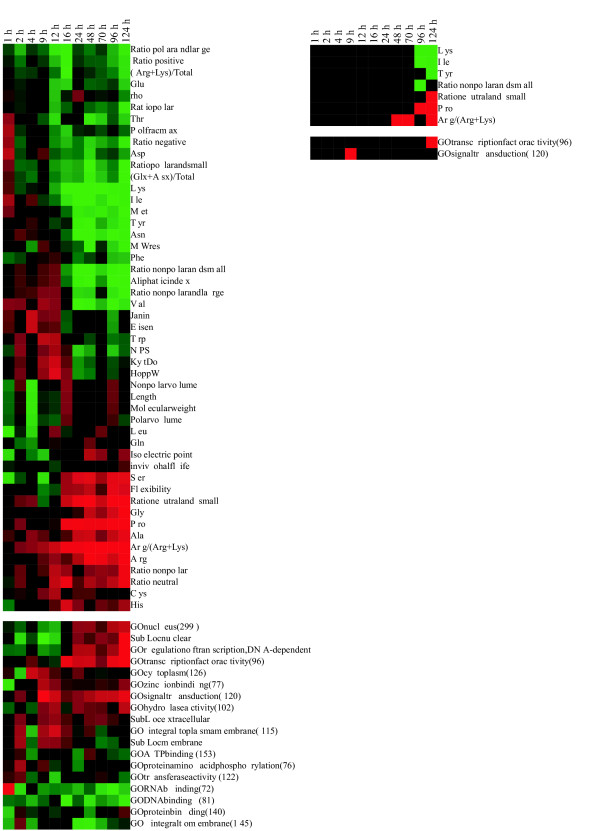
**Data analysis for human B cell differentiation**. (Left) Correlation coefficients for 1,358 genes involved in maturation in Ramos B cells at 11 sequential time points. (Right) Only the highly significant cases are shown.

Proline and R/(R+K) were significantly enriched during several consecutive late time points, and neutral and small residues were enriched in the last time point (Fig. 6), whereas I, K, Y; and nonpolar and small residues were underrepresented at all late time points. Among the gene ontologies, only transcription factor activity and signal transduction were significantly positively correlated, both at one time point. None of the protein subcellular localization predictions correlated significantly. Maximum correlations are generally seen at late time points, concurrent with the largest alterations in gene expression. This dataset yielded the least amount of significant findings, which is most likely due to the smaller number of genes in the dataset.

### *S. cerevisiae *cell cycle data analysis

Microarrays have been used to study gene expression in *S. cerevisiae *cultures synchronized by three independent methods: α factor arrest, elutriation, or arrest of a CDC15 temperature-sensitive mutant [22]. Expression of several yeast genes is known to vary periodically during the cell cycle, and the corresponding gene products may be involved in processes occurring only once during the cycle. To investigate these genes, cell cultures must be synchronized so that the cells are simultaneously in the same cell cycle stage. Several approaches are available for synchronization, three of which were used in this study. The first was elutriation whereas other two were cyclin-dependent. In addition, the effects of the G1 cyclin, Cln3p, and the B-type cyclin, Clb2p, were tested. Cyclins are special cell cycle regulators. Note that the CDC28 data was obtained from ref [34].

**Figure 7:**
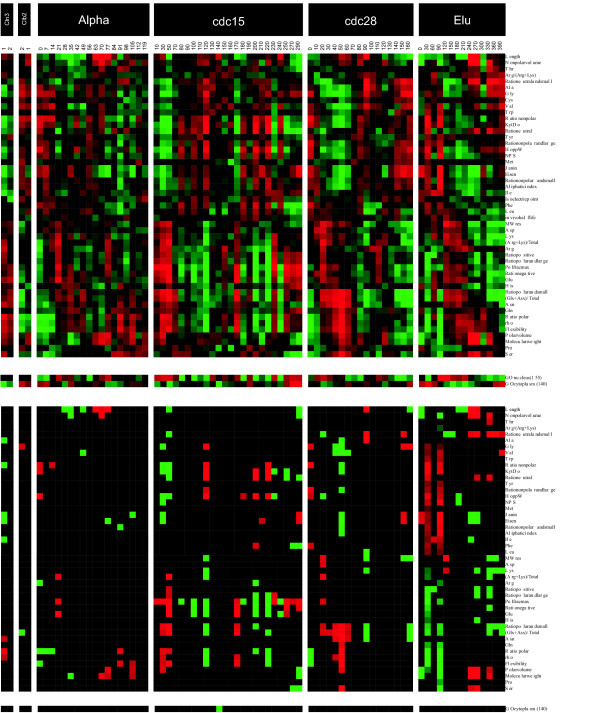
**Data analysis for the yeast cell cycle**. (Top) Correlation coefficients for 590 yeast genes in separate experiments. (Bottom) Only significant correlations are shown.

Several significant correlations are apparent in Fig. 7. Almost all the investigated sequence-based parameters covary with gene expression, but correlations at adjacent time points are rare. At some time points, there are no significant correlations, a recognizable difference compared with the other datasets. This leads to patchy patterns of covariation. If we ignore the time points for which there were few or no correlations, we notice that the different cell culture synchronization treatments yielded rather similar patterns. In the elutriation data, the positively correlated parameters are stronger at the beginning of the time series. Another feature typical for the yeast data is that the correlations are stable in the sense that they seldom change from positive to negative (or vice versa) during a treatment.

At the early time points, the correlations are positive (especially in the elutriation treatment) for F, G, I, L, V, W, Y, hydropathy values, as well as for ratios of nonpolar and large, nonpolar, and neutral amino acids. Correlations are mainly negative for E, K, (R+K)/total, rho, and ratios of positive, and polar and small residues. The correlation of V to gene expression is consistent with a previous yeast study [7]; however, since we analyzed dynamic changes, amino acids may appear enriched or underrepresented depending on the time point and the effectors.

Large numbers of correlations occur in the CDC15 and CDC28 experiments, in which the largest fraction of significant MIPS functional classifications was observed in a previous study [35]. This implies that there is consistency among the observations regarding physicochemical properties of proteins and their functional attributes in association with gene expression. Only one gene ontology class, cytoplasm, shows significant correlation – at just one time point. Presumably, the reason for the low abundance of significantly correlated ontologies lies in the smaller number of significantly altered genes compared with the other datasets.

### *D. melanogaster *life cycle data analysis

The *D. melanogaster *study was performed to follow development and gene expression in a multicellular model organism. In this dataset [23], the transcriptional profiles were investigated throughout the life cycle, from fertilization to aging adults. Samples of both males and females were taken during a complete time course of development of wild type fruit flies up to 30 days of adulthood.

**Figure 8:**
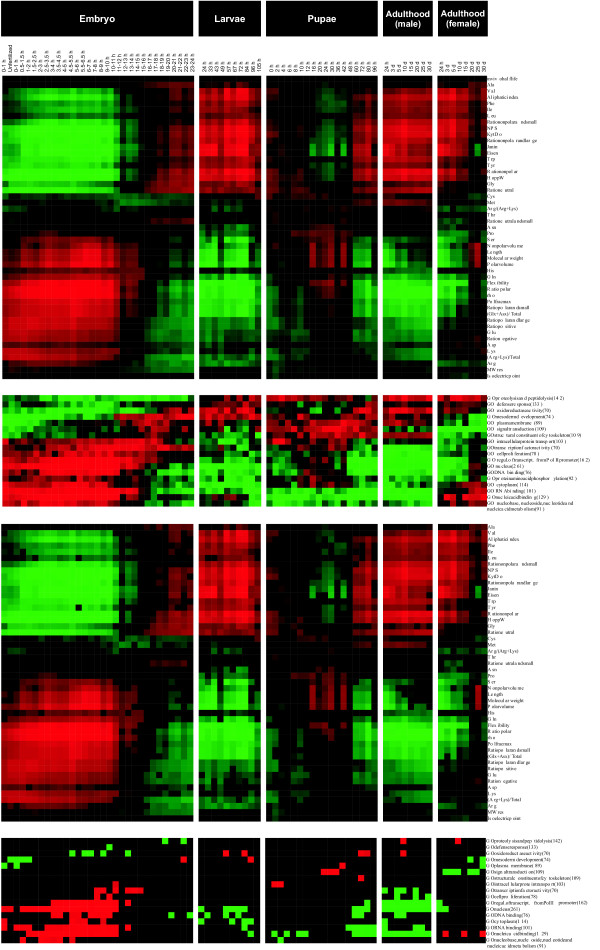
**Data analysis of the *D. melanogaster *life cycle**. (Top) Correlation coefficients of 2,976 genes in wild-type flies examined at 66 time points beginning at fertilization and spanning the embryonic, larval, and pupal periods as well as the first 30 days of adulthood. (Bottom) The most significant cases are shown. Two transition periods are apparent from the correlations. The continuity and strength of the correlations are unique to the *D. melanogaster *data.

There are dynamic correlation patterns between gene expression and proteome properties during the embryonic period and especially the pupae period (Fig. 8). Correlations are relatively stable in larva as well as during adulthood, especially in males. Continuity and strength of the correlations are unique to the *D. melanogaster *data, in that once a significant correlation occurs it remains for a prolonged period (i.e., over several consecutive time points).

The data contain two major transitions of correlations that do not coincide with developmental stages (Fig. 8). The first transition occurs during the embryo stage, from approximately 11 to 20 h, whereas the other occurs during the pupae period, from 4 to 48 h. There is a somewhat more stable period between 12 to 36 h. Interestingly, covariation patterns are very similar between the transition periods. The observed significant correlations from 15–16 h from the embryo stage until the end of the larval stage are quite similar to the time period from 60 h (pupae) through adulthood (especially in males). On the other hand, the pattern from the beginning of the embryonic stage to 10 h has a covariation pattern that is opposite to the two other conserved time periods. Those parameters having positive correlation at the beginning of the embryonic stage are negatively correlated at late embryo/larvae and late pupae/adult time points, and vice versa. If we associate the covariation patterns between proteome and gene expression during the early embryonic and larval periods to growth and differentiation, respectively, we can infer that pupae have a mixture of both patterns, reflecting a transition from growth to differentiation and then back to growth. Only a few significant correlations are apparent during the transitions, suggesting that the gene expression profile is not in balance (i.e., it changes from one stage to another).

The larval and adulthood data show stable and constant patterns, in which correlations occur between gene expression and a large number of parameters, including a negative correlation for charged and polar residues, their ratios, Polfrac max, rho and flexibility. Positive correlations are seen for aliphatic, aromatic and hydrophobic amino acids, aliphatic index, and hydropathy parameters. Contrary to the observations for T cells, I and L show similar trends. As noted above, the patterns of positive and negative correlations are almost mirror images when comparing the first half of the embryo stage with the two constant regions (i.e., late embryo and larval time points or the late pupae and adult period). Almost all protein properties have statistically significant correlations; the exceptions are N, T and the ratio of neutral and small residues, for which significant correlations occur sporadically.

Several gene ontologies (17 classes) have significant correlations. Six ontologies are related to gene expression/DNA binding. They all have positive correlations during the first half of the embryonic stage and negative correlations thereafter. Nuclear localization follows the same pattern. The correlation motifs for the other ontologies are less clear. Oxidoreductase activity correlates negatively at several time points during the embryonic stage and correlates positively at other time points throughout the experiment. The signal transduction ontology has a similar overall pattern, but only in the pupa and adult females.

## Conclusion

Strong correlations between transcriptome and proteome characteristics appear in all the datasets we studied. One could not expect to obtain similar results from the different studies, which differed with respect to both the processes (growth, differentiation, stimulation, and cell cycle) and proteins produced. The strength of the correlations is apparently associated with the intensity of gene expression. Thus, proteins having significantly altered expression do not have a random distribution of physicochemical properties. In particular, the yeast and *D. melanogaster *life cycle datasets have obvious recurring cycles of enrichment and underrepresentation of different properties. The magnitudes of underrepresentation and enrichment are proportional to gene expression in all experiments. These observations along with our findings related to the functional and structural properties of proteins as well as MIPS classification in earlier studies [7,21] suggest that protein characteristics, including function, structure, subcellular location, and physicochemical properties, are closely associated with gene expression. Furthermore, our method clearly detects dynamic shifts in the gene expression profiles, as exemplified in the *D. melanogaster *dataset.

Similar, although not identical correlations were obtained when analyzing time series datasets for human T and B cells, yeast, and *D. melanogaster*, indicating that the dynamic correlations between proteome-related parameters and gene expression likely represent a general paradigm. Since the association is strong and is observed at many levels, cells and organisms, and appears to be a widespread phenomenon, it likely reflects fundamentally important biological processes.

Only a few studies of system-wide transcriptome-proteome correlations have been published, including human heart [36] and platelets [37], mouse liver and kidney [38], and mosquito salivary gland [39]. Only functional features, mainly gene ontologies, were investigated in these articles. Certain ontologies were clearly enriched in all the cases. There was statistically significant colocalization of coexpressed genes in the mouse assay.

Proteome-wide isoelectric points and molecular masses, the two properties used for separation in 2D gels, have been analyzed for 103 organisms, including bacteria, archae and eukaryotes [40]. The comparison of properties of theoretical proteomes for 11 bacteria to the usage of 95 different carbon sources indicated that the ecological niche of bacteria correlates with their proteome parameters [40]. Comparable to these organism-wide, macro-level correlations, we observed several micro-level (time point) correlations in the different datasets of our present study.

There are presumably several reasons for the observed enrichment/depletion of protein properties, which might be related, for example, to cellular processes involved in changes in metabolism and signaling, localization of proteins within cells and compartments, and complexes and interactions formed between proteins. There are indications for the organellar enrichment of proteins having certain types of properties; for example, the need for positive charge in DNA-binding proteins (including histones). In certain disease conditions, the overall amino acid composition of proteins changes in cells, organs, or body fluids [41,42]. Thus, proteome parameters might have diagnostic value and may indicate that health and disease states are linked to both the production and properties of expressed proteins.

## Methods

Gene expression data were derived from peripheral T cells subjected to seven treatments: mock (untreated) cells; CD3- or CD28-stimulated cells; CD3 and CD28-costimulated cells; and cells treated with ionomycin along with phorbol 12-myristate 13-acetate (PMA), phytohemagglutinin, or FK506. Data were acquitted at six time points up to 48 hr [20]. We did not investigate the chemical treatment results in detail because they were quantitatively similar to CD3/CD28 co-treatment. Because there were only a few genes that had a significant change in expression (>1.5 fold) in the mock and CD28-stimulated datasets, we focused exclusively on the CD3-stimulated and CD3/CD28-costimulated datasets. In total, 4,359 cDNA elements (of about 18,000 genes and ESTs on the chip) representing 2,926 genes were significantly altered after CD3 stimulation or CD3/CD28 costimulation [20]. The data were taken from .

The B cell dataset for genes involved in maturation in anti-immunoglobulin M-stimulated Ramos cells indicated that close to 1,500 genes had significantly altered expression, at least at one time point [8,21]. These data were from our own experiments. In the *D. melanogaster *microarray data set, the transcriptional profiles were investigated throughout the life cycle [23]. RNA expression levels of 4,028 genes in wild-type flies were examined at 66 time points. Expression of 3,483 out of 4,028 (86%) changed significantly [P < 0.001; analysis of variance (ANOVA) during the 40-day survey period [23]. The data for genes were obtained from  from file Arbeitman.SOMtables.zip. Gene expression information was from .

A total of about 600 genes had altered gene expression patterns in yeast cultures synchronized by four independent methods (α factor arrest, elutriation, cdc28, and arrest of a cdc15 temperature-sensitive mutant) [22]. We also studied the effect of treatment with either the G1 cyclin, Cln3p, or the B-type cyclin, Clb2p. The data were collected from  and .

### Data mining

We used numerous bioinformatics methods to filter and merge information regarding gene and protein annotations in a number of databases and further calculated and predicted a large number of characteristics for each gene/protein. A dedicated database (Siermala et al., unpublished data) constructed for gene and protein information was extensively used for annotations and sequence identification. To identify the corresponding proteins, we used Locus Link ID numbers of ESTs and genes to retrieve UniProt sequences. FlyBase [43] was used to identify *D. melanogaster *protein sequences, and NCBI genome sequences were used for yeast.

### Sequence-derived variables

Physicochemical parameters and amino acid proportions were directly calculated from protein sequences. Amino acids were further investigated by analyzing the proportions of different groups of residues, namely positive (R, H, K), negative (D, E), neutral (A, N, C, Q, G, I, L, M, F, P, S, T, W, Y, V), polar (D, E, H, K, N, Q, R, S, T, Y), and nonpolar (A, C, F, G, I, L, M, P, V, W). Combinations of characteristics were also investigated, namely neutral and small (A, G, P, S, T), nonpolar and large (F, W, Y), nonpolar and small (I, L, M, V), polar and large (H, K, R), polar and small (D, E, N, Q). In addition, the ratio of Glx (E, Q) and Asx (D, N) to total amino acids, the ratio of R to R plus K, and the ratio of R and K to total amino acids were calculated.

Several parameters for physicochemical features described in the literature were calculated. The aliphatic index is defined as the relative volume occupied by aliphatic side chains (i.e., A, I, L, and V) and was calculated as follows

AI = X(Ala) + 2.9 × X(Val) + 3.9 × (X(Ile) + X(leu)),

where X(Ala), X(Val), X(Ile), and X(Leu) are mole percents for the amino acids [44]. Residues were classified as polar and non-polar as per Fisher's definition [45]. The volumes of each category were calculated by summing the products of the number of each residue [46]. *rho *is the ratio of polar to non-polar volumes. Nonpolar side chains (NPS), the frequency of nonpolar side chains, was calculated according to Waugh's definition by counting W, N, T, F, P, L, and V residues and expressing the sum as a fraction of the total number of residues [47]. MWRES is the molecular weight per residue. The average hydrophobicity of proteins [46] was calculated from:



where *n_i _*is number of residue *i*, and *H*_*i *_is the hydrophobicity value of the residue. Four different hydropathy scales [48-51] were used.

POLFRAC_MAX (polar fraction) in an extended chain conformation is calculated as:



where *NRES *is the total number of residues in the protein and POLFRAC_MAX is in units of e, the electronic charge, SURF is maximal accessible surface area, and POLSURF is polar surface area [52]. Molecular weight was calculated from the sequence as well as the number of amino acids. Isoelectric point was calculated with the EMBOSS  program iep. *In vivo *half-life of proteins was calculated according to [53].

Average flexibility [54,55] for each protein was predicted from the amino acid sequence using a 9-residue sliding window averaging technique with the formula:



where  and B_nc _is the flexibility parameter of the residue in position *i*.

For the T cell experiment [20], we identified 1,687 amino acid sequences for 2,926 genes. Sequences were found for 761 proteins in human B cell data and 415 for the yeast dataset. For *D. melanogaster *[23], 2,976 amino acid sequences for 4,028 genes/ESTs with significant changes in expression were identified and included in the analysis. The actual number of proteins used in any individual analysis varied due to different amounts of missing data in different experiments and at different time points.

### Subcellular localization

Subcellular localization of proteins was predicted using SubLoc software [56], which yielded predictions for four categories (cytoplasmic, nuclear, mitochondrial, and extracellular). Transmembrane regions were further predicted using the TMHMM server [57]. To assign each protein to a compartment, we used the SubLoc reliability index and the length of the predicted transmembrane region. If the value of the SubLoc prediction parameter was ≥ 5, the assignment was accepted for the most accurate prediction. In cases where the parameter was between 2 and 4, the length of the transmembrane stretch(es) was taken into account. If the transmembrane region was longer than 36, the protein was predicted to be membrane associated. In cases where the SubLoc prediction value was 1 and the transmembrane region was ≥ 18 residues (the average length of a transmembrane α-helix), the protein was predicted to be membrane spanning. We made predictions in T cell data for 443 proteins, 177 of which were transmembrane, 167 nuclear, 67 extracellular, 7 mitochondrial, and 25 cytoplasmic. Predictions were made for 224 proteins in the B cell data.

### Gene ontology

Gene ontology [26] information was extracted from Entrez Gene [58] or from annotated genome data for yeast sequences. In total, we identified the cell component ontology for 2,448 proteins, molecular function for 2,760 proteins, and biological processes for 2,748 proteins in the T cell dataset. Ontologies were available for 1,260 entries in the B cell dataset, for 590 in the yeast dataset, and for 2,293 genes/ESTs in the fruit fly dataset.

### Structural description

Hidden Markov Models (HMMs), downloaded from Superfamily [59], were used to search SCOP-derived domains [27] against the protein sequences in T cell data using the program HMMER [60], setting the e-value to <10e^-6 ^as a limit. SCOP information was available for 2,022 entries. The limit of e-values was set so that false positives were unlikely. Moreover, we compared a number of findings with those acquired using the InterProScan sequence searching service [61], which yielded nearly identical results.

### Correlation analysis

To monitor dynamic covariations between a number of proteome parameters and gene expression levels along the time series, we applied the Spearman linear correlation test. The schema of the analysis is presented in Fig. 1. The value of the correlation coefficient is dependent on the number of genes/proteins. A value of 0.12 was used for the B cell, T cell, and yeast datasets, and 0.3 was used for the much larger *Drosophila *dataset. To estimate the significance of the observations, a permutation test was performed. One-thousand permutations were calculated for each parameter at each time point in the B cell, T cell, and yeast datasets, and 100 permutations were calculated in the *D. melanogaster *dataset. To be regarded as significant, the p value had to be ≤ 0.05, both in the correlation and permutation calculations.

For TOC, the expression of genes at each time point are separately clustered. For the actual clustering, we used the SOM method, which organized the clusters according to the shapes of the expression profiles of genes. During the method development, we also monitored the clustering process visually; SOMs served as ideal visualization tools. Following TOC, the Spearman correlation was used to test the linear correlations between the medians of gene expression and protein variables in the corresponding clusters.

TOC was an essential step in facilitating the analysis of functional and structural attributes (ontology, subcellular localization, SCOP) because, due to the nature (binary and categorical) and scarceness of some of these data, a clustering measure was required before conducting the correlation test. Furthermore, all correlation coefficients could be compared by unifying the sample sizes with a fixed number of clusters (20) for each of the different types and data samples.

Since the functional and structural information variables were categorical (e.g., a hierarchy of ontology), we conducted correlation tests between the medians of the clusters of expression levels and medians of the ratios (observed/expected in each cluster) of the sequences attributed to the functional or structural variables. The expected number of a given ontology, subcellular localization, or structural variable for each cluster was calculated based on the size of the cluster and the total number of occurrences of the variable.

The correlation coefficients and p values for correlation and permutation tests are in Additional files 1, 2, 3, 4, 5 (CorrelationDataNumbersTCELLCD3.xls, CorrelationDataNumbersTCELLBCDs.xls, CorrelationDataNumbersBCELL.xls, CorrelationDataNumbersYEAST.xls, CorrelationDataNumbersDROSOPHILA.xls) for CD3-induced T cells, CD3/CD28-costimulated T cells, B cells, yeast, and *Drosophila *datasets, respectively.

### Visualization

To provide an intelligible report on the very large correlation analyses, we introduced a new type of visualization. Correlation matrices were formed in which columns and rows represent time points and variables, respectively. Each cell represents a correlation coefficient (theoretically ranging from -1 to 1) between the expression levels of the time point and the values of the variable. Red and green represent positive and negative correlations, respectively. The intensities of the colors are relative to the absolute value of the correlation coefficients. A significant red-colored proteome variable implies an increase in production (i.e., overexpression of proteins either attributed to a property concerning functional or structural variables [GO, SCOP, and SubLoc] or containing higher proportions of a property [amino acids and physicochemical parameters]) compared with the changes in expression of other proteins, which implies the enrichment of the property at that time point. Likewise, a significant green-colored variable implies a reduction in the production of proteins either associated with or containing lower proportions of the property (i.e., underrepresentation of the property at the time point). To determine which variables covary in a similar manner with expression levels over time, the matrix was clustered using SOMs.

### Tools and software

Script from CPAN  was used for clustering. We developed the software for all other analyses and calculations as well as for all visualizations.

## Authors' contributions

MTAS participated in the statistical analysis, preliminary program development, and drafted the manuscript. MS carried out the data mining and statistical analysis, developed computer tools, and drafted the manuscript. TOL participated in data analysis and developed data mining, analysis and visualization tools. MV conceived of the study, participated in its design and coordination, and drafted the manuscript. All authors read and approved the final manuscript.

## Supplementary Material

Additional File 1 CD3-stimulated T cells Click here for file

Additional File 2 CD3/CD28-costimulated T cells Click here for file

Additional File 3 B cells Click here for file

Additional File 4 Synchronized yeast cells Click here for file

Additional File 5 *Drosophila *life cycle dataset Click here for file

## References

[B1] Slonim DK (2002). From patterns to pathways: gene expression data analysis comes of age. Nat Genet.

[B2] Niehrs C, Pollet N (1999). Synexpression groups in eukaryotes. Nature.

[B3] Cohen BA, Mitra RD, Hughes JD, Church GM (2000). A computational analysis of whole-genome expression data reveals chromosomal domains of gene expression. Nat Genet.

[B4] Caron H, van Schaik B, van der Mee M, Baas F, Riggins G, van Sluis P, Hermus MC, van Asperen R, Boon K, Voute PA, Heisterkamp S, van Kampen A, Versteeg R (2001). The human transcriptome map: clustering of highly expressed genes in chromosomal domains. Science.

[B5] Brazma A, Jonassen I, Vilo J, Ukkonen E (1998). Predicting gene regulatory elements in silico on a genomic scale. Genome Res.

[B6] Roth FP, Hughes JD, Estep PW, Church GM (1998). Finding DNA regulatory motifs within unaligned noncoding sequences clustered by whole-genome mRNA quantitation. Nat Biotechnol.

[B7] Jansen R, Gerstein M (2000). Analysis of the yeast transcriptome with structural and functional categories: characterizing highly expressed proteins. Nucleic Acids Res.

[B8] Ollila J, Vihinen M (2002). Microarray analysis of B-cell stimulation. Vitam Horm.

[B9] Doniger SW, Salomonis N, Dahlquist KD, Vranizan K, Lawlor SC, Conklin BR (2003). MAPPFinder: using Gene Ontology and GenMAPP to create a global gene-expression profile from microarray data. Genome Biol.

[B10] Draghici S, Khatri P, Bhavsar P, Shah A, Krawetz SA, Tainsky MA (2003). Onto-Tools, the toolkit of the modern biologist: Onto-Express, Onto-Compare, Onto-Design and Onto-Translate. Nucleic Acids Res.

[B11] Zeeberg BR, Feng W, Wang G, Wang MD, Fojo AT, Sunshine M, Narasimhan S, Kane DW, Reinhold WC, Lababidi S, Bussey KJ, Riss J, Barrett JC, Weinstein JN (2003). GoMiner: a resource for biological interpretation of genomic and proteomic data. Genome Biol.

[B12] Ge H, Liu Z, Church GM, Vidal M (2001). Correlation between transcriptome and interactome mapping data from Saccharomyces cerevisiae. Nat Genet.

[B13] Kemmeren P, van Berkum NL, Vilo J, Bijma T, Donders R, Brazma A, Holstege FC (2002). Protein interaction verification and functional annotation by integrated analysis of genome-scale data. Mol Cell.

[B14] Fukuchi S, Nishikawa K (2001). Protein surface amino acid compositions distinctively differ between thermophilic and mesophilic bacteria. J Mol Biol.

[B15] Cedano J, Aloy P, Perez-Pons JA, Querol E (1997). Relation between amino acid composition and cellular location of proteins. J Mol Biol.

[B16] Drawid A, Jansen R, Gerstein M (2000). Genome-wide analysis relating expression level with protein subcellular localization. Trends Genet.

[B17] Hegyi H, Gerstein M (1999). The relationship between protein structure and function: a comprehensive survey with application to the yeast genome. J Mol Biol.

[B18] Andrade MA, O'Donoghue SI, Rost B (1998). Adaptation of protein surfaces to subcellular location. J Mol Biol.

[B19] Tekaia F, Yeramian E, Dujon B (2002). Amino acid composition of genomes, lifestyles of organisms, and evolutionary trends: a global picture with correspondence analysis. Gene.

[B20] Diehn M, Alizadeh AA, Rando OJ, Liu CL, Stankunas K, Botstein D, Crabtree GR, Brown PO (2002). Genomic expression programs and the integration of the CD28 costimulatory signal in T cell activation. Proc Natl Acad Sci U S A.

[B21] Ollila J, Vihinen M (2003). Stimulation-induced gene expression in Ramos B-cells. Genes Immun.

[B22] Spellman PT, Sherlock G, Zhang MQ, Iyer VR, Anders K, Eisen MB, Brown PO, Botstein D, Futcher B (1998). Comprehensive identification of cell cycle-regulated genes of the yeast Saccharomyces cerevisiae by microarray hybridization. Mol Biol Cell.

[B23] Arbeitman MN, Furlong EE, Imam F, Johnson E, Null BH, Baker BS, Krasnow MA, Scott MP, Davis RW, White KP (2002). Gene expression during the life cycle of Drosophila melanogaster. Science.

[B24] Mustelin T, Tasken K (2003). Positive and negative regulation of T-cell activation through kinases and phosphatases. Biochem J.

[B25] Viola A, Schroeder S, Sakakibara Y, Lanzavecchia A (1999). T lymphocyte costimulation mediated by reorganization of membrane microdomains. Science.

[B26] Ashburner M, Ball CA, Blake JA, Botstein D, Butler H, Cherry JM, Davis AP, Dolinski K, Dwight SS, Eppig JT, Harris MA, Hill DP, Issel-Tarver L, Kasarskis A, Lewis S, Matese JC, Richardson JE, Ringwald M, Rubin GM, Sherlock G (2000). Gene ontology: tool for the unification of biology. The Gene Ontology Consortium. Nat Genet.

[B27] Andreeva A, Howorth D, Brenner SE, Hubbard TJ, Chothia C, Murzin AG (2004). SCOP database in 2004: refinements integrate structure and sequence family data. Nucleic Acids Res.

[B28] Akashi H, Gojobori T (2002). Metabolic efficiency and amino acid composition in the proteomes of Escherichia coli and Bacillus subtilis. Proc Natl Acad Sci U S A.

[B29] Greenbaum D, Jansen R, Gerstein M (2002). Analysis of mRNA expression and protein abundance data: an approach for the comparison of the enrichment of features in the cellular population of proteins and transcripts. Bioinformatics.

[B30] Anderson L, Seilhamer J (1997). A comparison of selected mRNA and protein abundances in human liver. Electrophoresis.

[B31] Gygi SP, Rochon Y, Franza BR, Aebersold R (1999). Correlation between protein and mRNA abundance in yeast. Mol Cell Biol.

[B32] Nel AE (2002). T-cell activation through the antigen receptor. Part 1: signaling components, signaling pathways, and signal integration at the T-cell antigen receptor synapse. J Allergy Clin Immunol.

[B33] Nel AE, Slaughter N (2002). T-cell activation through the antigen receptor. Part 2: role of signaling cascades in T-cell differentiation, anergy, immune senescence, and development of immunotherapy. J Allergy Clin Immunol.

[B34] Cho RJ, Campbell MJ, Winzeler EA, Steinmetz L, Conway A, Wodicka L, Wolfsberg TG, Gabrielian AE, Landsman D, Lockhart DJ, Davis RW (1998). A genome-wide transcriptional analysis of the mitotic cell cycle. Mol Cell.

[B35] Gerstein M, Jansen R (2000). The current excitement in bioinformatics-analysis of whole-genome expression data: how does it relate to protein structure and function?. Curr Opin Struct Biol.

[B36] Ruse CI, Tan FL, Kinter M, Bond M (2004). Intregrated analysis of the human cardiac transcriptome, proteome and phosphoproteome. Proteomics.

[B37] McRedmond JP, Park SD, Reilly DF, Coppinger JA, Maguire PB, Shields DC, Fitzgerald DJ (2004). Integration of proteomics and genomics in platelets: a profile of platelet proteins and platelet-specific genes. Mol Cell Proteomics.

[B38] Mijalski T, Harder A, Halder T, Kersten M, Horsch M, Strom TM, Liebscher HV, Lottspeich F, de Angelis MH, Beckers J (2005). Identification of coexpressed gene clusters in a comparative analysis of transcriptome and proteome in mouse tissues. Proc Natl Acad Sci U S A.

[B39] Ribeiro JM, Charlab R, Pham VM, Garfield M, Valenzuela JG (2004). An insight into the salivary transcriptome and proteome of the adult female mosquito Culex pipiens quinquefasciatus. Insect Biochem Mol Biol.

[B40] Knight CG, Kassen R, Hebestreit H, Rainey PB (2004). Global analysis of predicted proteomes: functional adaptation of physical properties. Proc Natl Acad Sci U S A.

[B41] Benga G, Ferdinand W (1995). Amino acid composition of rat and human liver microsomes in normal and pathological conditions. Biosci Rep.

[B42] Forli L, Pedersen JI, Vatn M, Kofstad J, Boe J, Bjortuft (2000). Serum amino acids in relation to nutritional status, lung function and energy intake in patients with advanced pulmonary disease. Respir Med.

[B43] (2003). The FlyBase database of the Drosophila genome projects and community literature. Nucleic Acids Res.

[B44] Ikai A (1980). Thermostability and aliphatic index of globular proteins. J Biochem (Tokyo).

[B45] Fisher HF (1964). A Limiting Law Relating the Size and Shape of Protein Molecules to Their Composition. Proc Natl Acad Sci U S A.

[B46] Bigelow CC (1967). On the average hydrophobicity of proteins and the relation between it and protein structure. J Theor Biol.

[B47] Waugh DF (1954). Protein-protein interactions. Adv Protein Chem.

[B48] Kyte J, Doolittle RF (1982). A simple method for displaying the hydropathic character of a protein. J Mol Biol.

[B49] Eisenberg D, Weiss RM, Terwilliger TC (1982). The helical hydrophobic moment: a measure of the amphiphilicity of a helix. Nature.

[B50] Janin J (1979). Surface and inside volumes in globular proteins. Nature.

[B51] Hopp TP, Woods KR (1981). Prediction of protein antigenic determinants from amino acid sequences. Proc Natl Acad Sci U S A.

[B52] Baumann G, Frommel C, Sander C (1989). Polarity as a criterion in protein design. Protein Eng.

[B53] Bachmair A, Finley D, Varshavsky A (1986). In vivo half-life of a protein is a function of its amino-terminal residue. Science.

[B54] Vihinen M (1987). Relationship of protein flexibility to thermostability. Protein Eng.

[B55] Vihinen M, Torkkila E, Riikonen P (1994). Accuracy of protein flexibility predictions. Proteins.

[B56] Hua S, Sun Z (2001). Support vector machine approach for protein subcellular localization prediction. Bioinformatics.

[B57] Krogh A, Larsson B, von Heijne G, Sonnhammer EL (2001). Predicting transmembrane protein topology with a hidden Markov model: application to complete genomes. J Mol Biol.

[B58] Maglott D, Ostell J, Pruitt KD, Tatusova T (2005). Entrez Gene: gene-centered information at NCBI. Nucleic Acids Res.

[B59] Gough J, Karplus K, Hughey R, Chothia C (2001). Assignment of homology to genome sequences using a library of hidden Markov models that represent all proteins of known structure. J Mol Biol.

[B60] McClure MA, Smith C, Elton P (1996). Parameterization studies for the SAM and HMMER methods of hidden Markov model generation. Proc Int Conf Intell Syst Mol Biol.

[B61] Mulder NJ, Apweiler R, Attwood TK, Bairoch A, Barrell D, Bateman A, Binns D, Biswas M, Bradley P, Bork P, Bucher P, Copley RR, Courcelle E, Das U, Durbin R, Falquet L, Fleischmann W, Griffiths-Jones S, Haft D, Harte N, Hulo N, Kahn D, Kanapin A, Krestyaninova M, Lopez R, Letunic I, Lonsdale D, Silventoinen V, Orchard SE, Pagni M, Peyruc D, Ponting CP, Selengut JD, Servant F, Sigrist CJ, Vaughan R, Zdobnov EM (2003). The InterPro Database, 2003 brings increased coverage and new features. Nucleic Acids Res.

